# Landscape of protein–small ligand binding modes

**DOI:** 10.1002/pro.2971

**Published:** 2016-07-04

**Authors:** Kota Kasahara, Kengo Kinoshita

**Affiliations:** ^1^ College of Life SciencesRitsumeikan UniversityKusatsuShiga525‐8577Japan; ^2^ Graduate School of Information SciencesTohoku UniversitySendaiMiyagi980‐8597Japan; ^3^ Tohoku Medical Megabank OrganizationTohoku UniversitySendaiMiyagi980‐8573Japan; ^4^ Institute of Development, Aging and Cancer, Tohoku UniversitySendaiMiyagi980‐8575Japan

**Keywords:** protein–ligand complexes, binding modes, molecular recognition, bioinformatics, chemoinformatics, database

## Abstract

Elucidating the mechanisms of specific small‐molecule (ligand) recognition by proteins is a long‐standing conundrum. While the structures of these molecules, proteins and ligands, have been extensively studied, protein–ligand interactions, or binding modes, have not been comprehensively analyzed. Although methods for assessing similarities of binding site structures have been extensively developed, the methods for the computational treatment of binding modes have not been well established. Here, we developed a computational method for encoding the information about binding modes as graphs, and assessing their similarities. An all‐against‐all comparison of 20,040 protein–ligand complexes provided the landscape of the protein–ligand binding modes and its relationships with protein‐ and chemical spaces. While similar proteins in the same SCOP Family tend to bind relatively similar ligands with similar binding modes, the correlation between ligand and binding similarities was not very high (*R* ^2^ = 0.443). We found many pairs with novel relationships, in which two evolutionally distant proteins recognize dissimilar ligands by similar binding modes (757,474 pairs out of 200,790,780 pairs were categorized into this relationship, in our dataset). In addition, there were an abundance of pairs of homologous proteins binding to similar ligands with different binding modes (68,217 pairs). Our results showed that many interesting relationships between protein–ligand complexes are still hidden in the structure database, and our new method for assessing binding mode similarities is effective to find them.

## Introduction

Elucidating the molecular mechanisms underlying the specific recognition of small‐molecules, or ligands, by proteins is a long‐standing conundrum in the field of protein science. Toward this goal, the wealth of three‐dimensional (3D) structure data of proteins deposited in the Protein Data Bank (PDB)^1^ provides fruitful insights, because the structure data of molecular complexes describe the atomic details of molecular interactions, which generate the specificity of ligand recognition. To understand the principles of this phenomenon, comprehensive analyses of a variety of protein–ligand interactions and extraction of the knowledge from the huge database are required. “Structural bioinformatics” has tackled these challenges.^2^, ^3^ In particular, the 3D structures of ligand‐binding sites, the protein surfaces contacting the ligand, have been extensively analyzed. Several statistical studies on binding site structures have revealed various structural motifs shared among evolutionally distant proteins.^4^, ^5^, ^6^, ^7^ The counterparts of the molecular interactions, meaning the ligands, have been investigated in another discipline called “chemoinformatics”. Several chemoinformatic techniques for characterizing and comparing the structures of chemical compounds as 1D vectors have been developed.^8^, ^9^

The database analyses by structural bioinformatics and chemoinformatics have revealed the landscapes of the protein structure space^10^ and the chemical space,^11^ respectively. Mapping these two spaces demonstrated that similar proteins do not always recognize similar ligands, and vice versa.^12^, ^13^ The mechanistic details of this nonlinear behavior of the relationships between these two spaces remain largely unclear. In order to fill the gap, the physical molecular interactions between proteins and ligands must be investigated.

Statistical studies of molecular interactions have mainly focused on the statistics of atomic interactions, meaning the atomic contacts between two atoms, groups, or small moieties of molecules, mediated by hydrogen bonds, van der Waals contacts, and π–π stacking.^14^, ^15^, ^16^, ^17^, ^18^ We previously reported statistical pattern analyses of the spatial distributions of ligand atoms contacting amino acid residues.^19^ These studies illuminated the propensities and patterns in the spatial configurations of interacting atoms. These atomic interactions can be considered as the building blocks of protein–ligand molecular interactions. In other words, a protein recognizes its ligand with a combination of atomic interactions. In spite of its importance, there is no gold standard for the computational analyses of the combinations, or the binding modes.

The “interaction fingerprint” method encodes a binding mode as a binary vector, with elements that reflect whether a residue interacts with a ligand using a particular type of interaction: e.g., hydrogen bonds and hydrophobic contacts.^20^ While this method is effective for postdocking analyses, it cannot be applied to compare binding modes between different proteins, because a sequence alignment is required for the comparison. Another technique, reported by Desaphy *et al*., is a universal method to compare protein–ligand binding modes, and it is effective for virtual screening tasks.^21^ This method encodes protein–ligand interactions as an integer vector or a complete graph, with information about pharmacophore types and distances between the interacting protein or ligand atoms (or their mid‐points). Their similarity measurement is highly correlated with the similarities of the binding sites and the ligand structures, because this graph explicitly includes information about the relative spatial positions of amino acids in the binding sites and the ligand atoms. This method simultaneously compares both molecular structures and interactions. However, as described above, numerous proteins and ligands display promiscuous properties, and thus similar structures do not always imply similar interactions. The current methods to encode protein or chemical structures cannot detect similar interactions between dissimilar proteins or ligands.

Here, we propose a new method for encoding information about binding modes and assessing their similarities. In this method, a binding mode is encoded as a bipartite graph, consisting of two kinds of nodes defining an amino acid residue or a ligand atom. Edges encode information about local interactions, defined as the relative positions of a contacting pair of an amino acid residue and a ligand atom. The relative positions between amino acids and those between ligand atoms were not considered. In order to encoding the relative positions of an interacting pair, we applied the statistical pattern recognition analysis method, presented in our previous study for labeling types of edges.^19^, ^22^ Using this method, we assessed the similarities of the binding modes for all pairs of 20,040 protein–ligand complexes, and compared them with the similarities of their protein structures and ligand structures. As a result, while similar proteins tend to bind similar ligands with similar binding modes, the correlations between ligands and binding similarities are not so high. We found many pairs of protein–ligand complexes with interesting relationships, such as pairs of dissimilar proteins and ligands with similar binding modes, and pairs of similar proteins and ligands with dissimilar binding modes.

## Results

### Method overview

We developed a graphical representation for the protein–ligand binding modes, named the “binding graph”, and a similarity measure between the binding graphs. The binding graph is a bipartite graph, consisting of two types of nodes that are ligand atoms or amino acid residues. The edges between them represent the atomic interactions between nodes with labels annotating the types of the interactions, which are defined by the statistical analysis of the atomic interactions in the dataset. Each 3D structure of a protein–ligand complex can be transformed into one binding graph. Using this graph, an all‐against‐all comparison of the binding mode similarities in our dataset was performed with the two other similarity measures; i.e., the SCOP classification^23^ and the fingerprint‐based chemical structure similarity of ligands.^24^ The binding similarity (*S*_b_) and ligand similarity (*S*_l_) are defined as real values from 0.0 to 1.0. The protein similarity (*S*_p_) is defined as the 5‐level accordance in the SCOP hierarchy; pairs with different Classes, the same Class, the same Fold, the same Superfamily, and the same Family. In addition, we also analyzed the identity of the binding sites in each pairs with the same Superfamily or Family.

**Figure 1:**
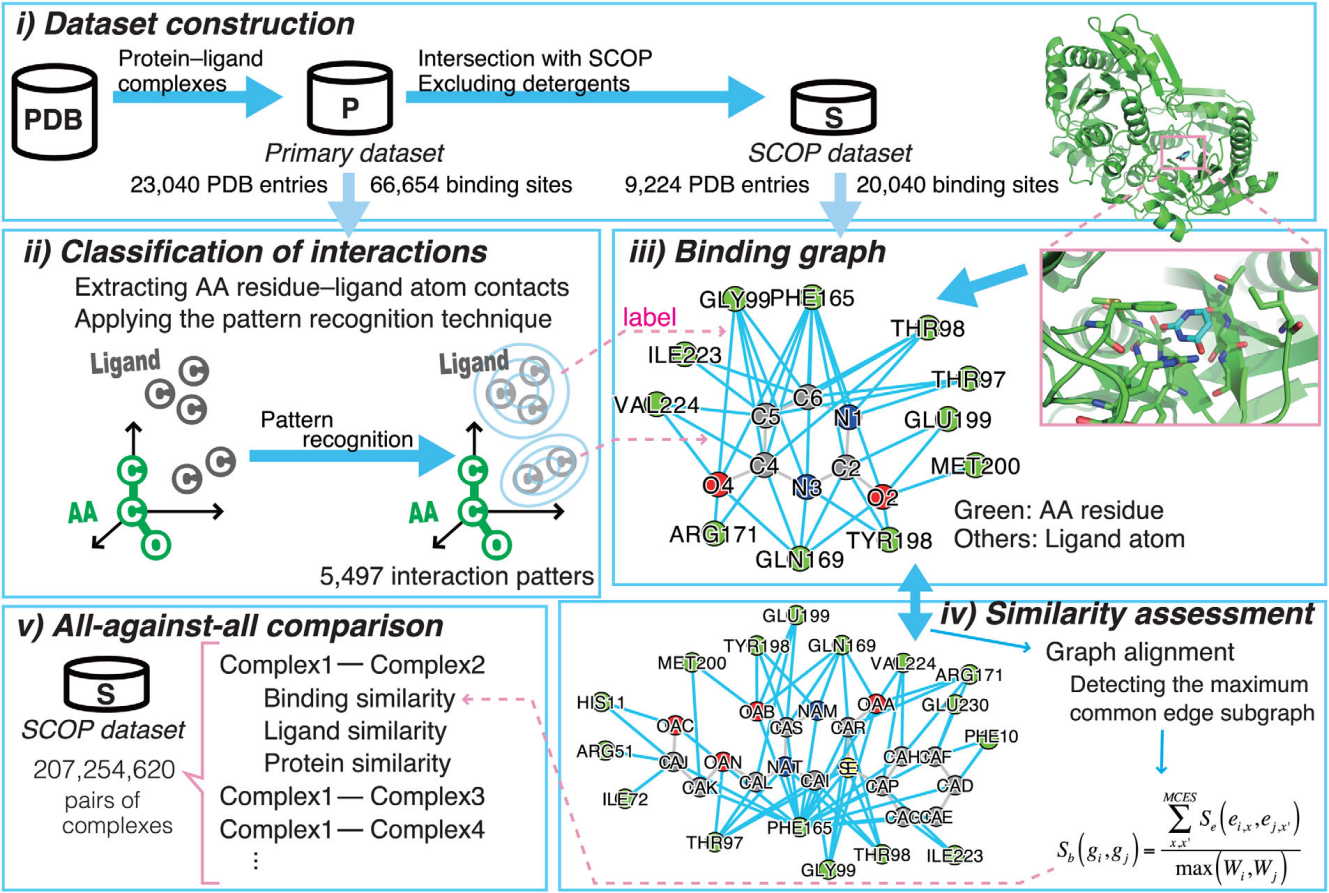
Method overview. The methods can be divided into five parts. (i) The two datasets of protein–ligand complexes, represented by the symbols “P” and “S”, were built. The “P”rimary dataset is a subset of the PDB, and the “S”COP dataset is a subset of the primary dataset. (ii) The patterns of interaction geometries observed in the primary dataset were defined. The green filled circles denote three covalently linked protein atoms (“AA” stands for amino acid). The axes of the local coordinate system, defined by the positions of the three atoms, are shown by the black arrows. The gray filled circles indicate the distribution of ligand atoms interacting with the protein atoms. By applying the pattern recognition technique, the distribution was fitted on to the Gaussian mixture model. Each Gaussian element is depicted as a set of concentric circles. The patterns defined here were used for labels in the next part. (iii) Each complex in the SCOP dataset was converted into the binding graph. The green circles indicate amino acid residues and other circles indicate ligand atoms. The cyan lines between them represent interactions with the patterns. (iv) The similarity between two binding graphs was assessed, based on the alignment of the two graphs. The details of this step are presented in Supporting Information Figure S1. (v) This similarity assessment was performed for all pairs of protein–ligand complexes in the SCOP dataset. In addition, the other two similarities; i.e., protein structure and ligand structure similarities, were also assessed.

The methods are summarized in Figure 1. The methods consist of the following five parts: (i) dataset construction, (ii) classification of protein–ligand atomic interactions, (iii) encoding binding modes of protein–ligand complexes as binding graphs, (iv) similarity assessments between two binding graphs (see also Supporting Information Fig. S1), and (v) all‐against‐all comparison of the protein–ligand complexes, using the three similarity measures; i.e., protein structures, ligand chemical structures, and binding modes.

### Dataset constructions and classification of atomic contacts

The “primary dataset” is composed of 23,040 PDB entries, including 49,361 polypeptide chains and 66,654 protein–ligand binding sites. This dataset is the same as the primary dataset used in our previous report.^19^ With the combination of 80 types of protein fragments and 25 types of ligand atoms, 80 × 25 = 2,000 types of interactions can exist. Since many of them are not observed or only rarely observed in the dataset, these types (<100 data points for each type) were removed, resulting in the consideration of only 955 types of interactions. For each type of interaction, the pattern recognition technique was applied and the spatial arrangements of the atomic interactions were classified, by fitting to the Gaussian mixture model. Each Gaussian function in the mixture models with ≥100 interactions in the dataset and the probability of existence (π_k_) ≥0.1 is referred as an “interaction pattern”. As a result, 5,497 interaction patterns were found in the dataset. An edge in the binding graphs (see “Methods”) was labeled with a set of interaction patterns. As a result, 44 out of 80 types of protein fragments, and 19 out of 25 types of ligand atoms, have at least one interaction pattern (Supporting Information Tables S1 and S2).

The SCOP dataset was obtained by taking the intersection between the primary dataset and SCOP ver. 1.75,^23^ and removing the major nonspecific ligands (Supporting Information Table S3) and the complexes with a small number of interactions (≤10 edges in the binding graph). This dataset is composed of 9,224 PDB entries, including 20,040 protein–ligand complexes, and 1,084 SCOP Families and 3,507 kinds of ligands. Details of the datasets are summarized in Supporting Information Table S4. The distributions of the numbers of nodes and edges in each binding graph are shown in Supporting Information Figure S2.

### Distribution of each type of similarity

We calculated the ligand and binding similarities, *S*_l_ and *S*_b_, for all pairs of complexes in the SCOP dataset. The average values of the binding and ligand similarities among all pairs are *μ*(*S*_b__,__all_) = 0.0420 and *μ*(*S*_l,all_) = 0.187, respectively. The values of the binding similarity tend to be lower than those of the ligand similarity, because our definition of *S*_b_ is based on the matching between two graphs consisting of edges attributed with highly sparse vectors; i.e., the 5,497‐dimensional vector ***w***_*i,n*_. Thus, the binding similarity metric is much more sparse than the ligand similarity space. As the large difference between the distributions of *S*_b_ and *S*_l_ are sometimes inconvenient for comparisons, the standardized score *Z*_b_ and *Z*_l_ are defined, as the following equation:

(1) $$Z=\frac{S-\mu }{\sigma },$$

where *Z* denotes the *Z*‐score (*Z*_b_ or *Z*_l_), *S* denotes the raw score (*S*_b_ and *S*_l_), *μ* and *σ* indicate the mean and the standard deviation of the raw score in the dataset (*μ*_b_ and *μ*_l_; *σ*_b_ and *σ*_l_).

**Figure 2:**
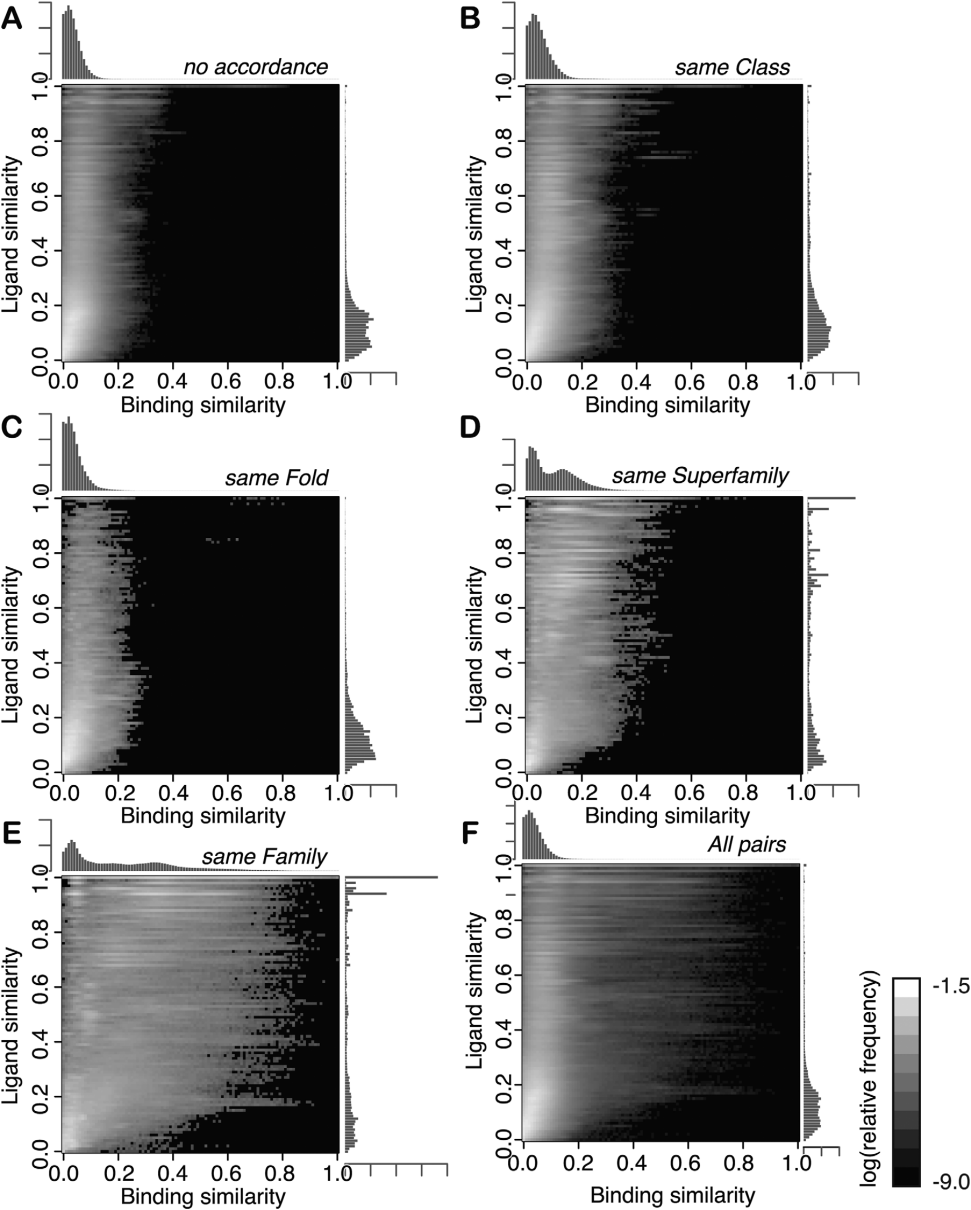
Distributions of ligand and binding similarities. The horizontal and vertical axes denote the binding and ligand similarities, respectively. The gray scale plot indicates the log frequency of the similarities. Brighter colors mean higher frequency, as shown in the scale bar on the right of the histogram. The bar plots along each axis are the distributions of each similarity. The values are the relative frequency (not the logged value). (A–E) The distributions in the pairs with no accordance in the SCOP hierarchy, the same Class, the same Fold, the same Superfamily, and the same Family, respectively. (F) The distribution in all pairs.

The 2D histogram of the relative frequency of pairs over ligand and binding similarities for all pairs is shown in Figure 2(F), and the histograms of the relative frequency of each similarity are shown along each axis of the 2D histogram. The *S*_b_ distribution peaks at *S*_b_ = 0.03, and the frequency monotonically decreases. The pairs with *S*_b_ ≥ 0.11 (*Z*_b_ ≥ 1.5) and *S*_b_ ≥ 0.18 (*Z*_b_ ≥ 3.2) are the top 5% and 1% most similar pairs, respectively. In the case of the ligand similarity, the distribution of *S*_l_ is broader and there is a plateau in the range from 0.08 to 0.20. There are some pairs at *S*_l_ = 1.0, which correspond to pairs of identical, frequently appearing ligands; e.g., mono‐nucleotides, sugars, and heme. The pairs with *S*_l_ ≥ 0.64 (*Z*_l_ ≥ 2.4) and *S*_l_ ≥ 0.96 (*Z*_l_ ≥ 4.1) are the top 5 and 1% most similar pairs of ligands, respectively. For the protein similarity, the ratio of pairs in the same Class, Fold, Superfamily, and Family are 33.9, 0.8, 1.0, and 0.9%, respectively. The remaining 63.5% lack any accordance in the SCOP hierarchy. The 2D histogram shows that there are no clear correlations between *S*_b_ and *S*_l_, and their *R* ^2^ value is 0.308. The horizontal bright streaks in the 2D histogram indicate that pairs with the same ligand similarity exhibit various binding mode similarities. This means that the binding mode similarity can diverge, even with the same ligand similarity. The distributions in each protein similarity show that similar proteins tend to bind similar ligands and employ similar binding modes [Fig. 2(A–E)]. In particular, the *S*_b_ shows the bi‐modal distributions in the same Superfamily and the same Family [Fig. 2(D,E)]. The second peak roughly corresponds to pairs of identical or very similar ligands in the same binding sites.

### Relationships among protein, ligand, and binding similarities

**Figure 3:**
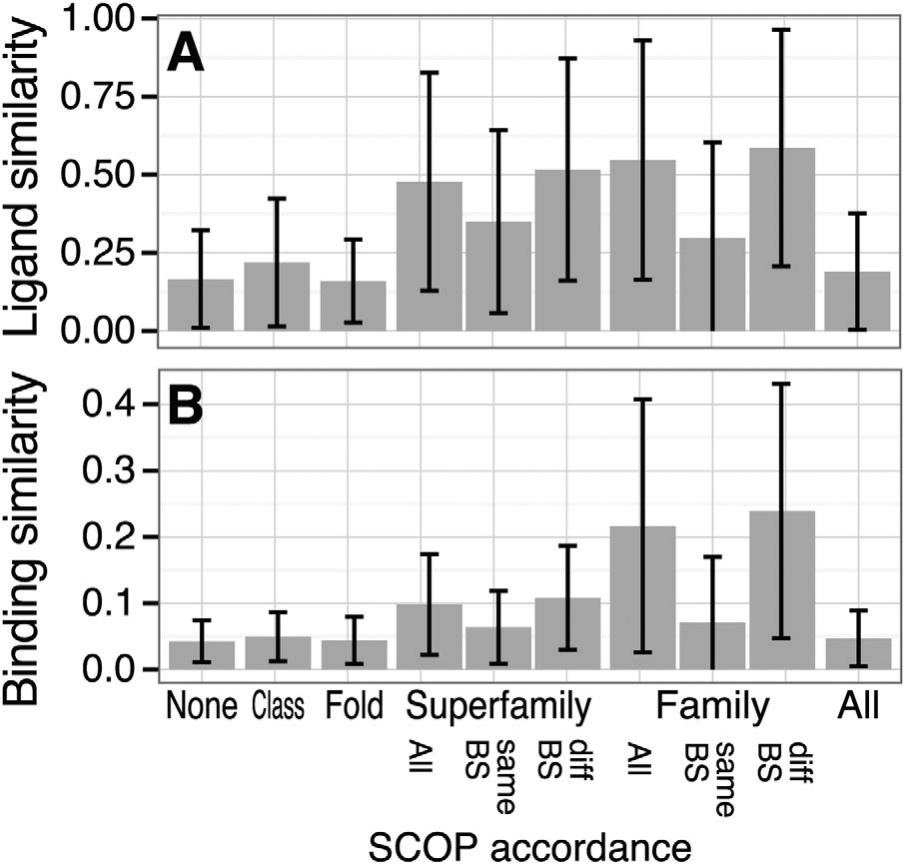
Ligand and binding similarities in each level of protein similarities. The average and standard deviation of (A) ligand and (B) binding similarities, in each level of protein similarity. “None” indicates the pairs in different Classes. “Total” indicates all pairs in the SCOP dataset.

The average and the standard deviations of *S*_b_ and *S*_l_ in each level of *S*_p_ are shown in Figure 3 and Supporting Information Table S1. While the SCOP dataset includes 10 Classes, 95.5% of the complexes are from one of the four major Classes: a, b, c, and d. Among all of the pairs of complexes, 63.5% of the pairs are with different Classes (the average similarities in each pair of Classes are shown in Supporting Information Fig. S3). Among the three lowest levels of *S*_p_ (proteins from different Superfamilies), there are no clear differences in both *S*_b_ and *S*_l_ [Fig. 3]. In contrast, for the pairs in the same Superfamily, the averages of *S*_b_ and *S*_l_ are 2.31‐ and 2.88‐times larger than those in different Classes. In the case of pairs in the same Family, they are 5.10‐ and 3.30‐times larger than those in different Classes. This result suggests that the accordance in Class and Fold levels does not necessarily imply the high similarity of ligands and their binding modes, and the proteins in the same Superfamily or Family are expected to have similar ligands or binding modes. While this trend is shared in both the ligand and binding similarities, the correlation of *S*_b_ and *S*_l_ is not very high. There are many pairs with dissimilar ligands and/or dissimilar binding modes even in the same Family.

In addition to the SCOP classification, we also analyzed the relationship between the protein sequence identity and the other similarities, for pairs within the same Superfamily (Supporting Information Fig. S4). The pairs with sequence identity ≥90% showed high ligand and binding similarities. For the other pairs (sequence identity <90%), there were no significant differences in the ligand and binding similarities, regardless of the sequence identity.

**Figure 4:**
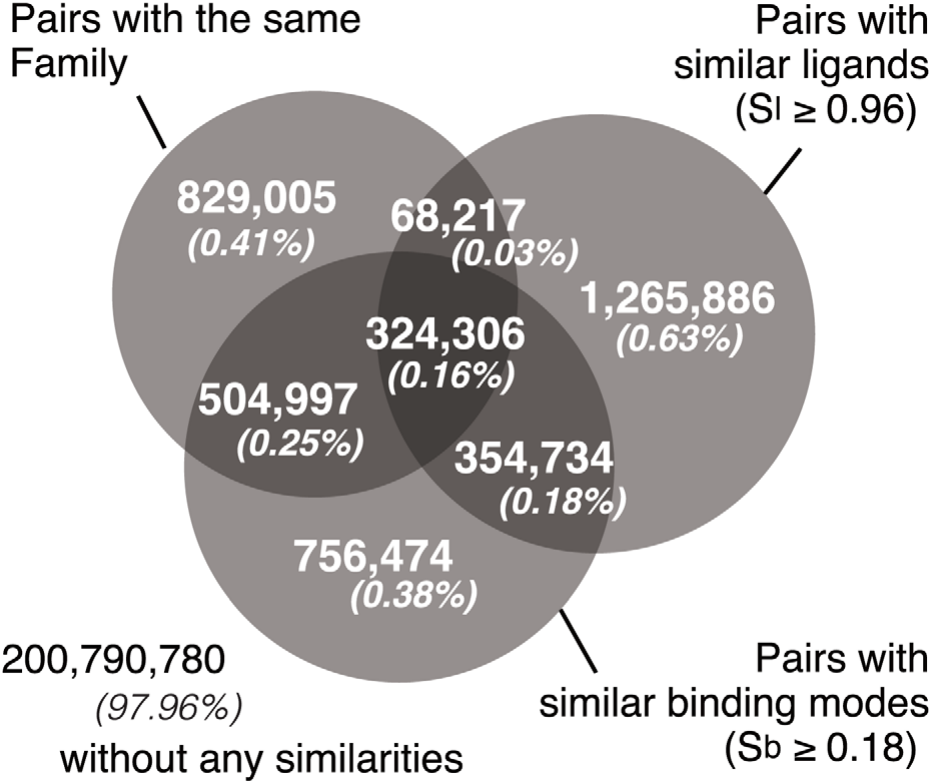
Number of similar pairs of complexes. The number of pairs in each combination of the three similarity measures is displayed as a Venn diagram.

For simplicity, the thresholds of the similarities in *S*_b_ and *S*_l_ are introduced, as the top 1% highest similarities. The pairs with *S*_b_ ≥ 0.18 (*Z*_b_ ≥ 3.2) or *S*_l_ ≥ 0.96 (*Z*_l_ ≥ 4.1) are considered as similar binding modes or similar ligands, respectively. For proteins, pairs with the same Superfamily are considered as similar proteins. The numbers of pairs in each combination of similarities are summarized as the Venn diagram in Figure 4, which shows that only limited regions are overlapped. 0.16% of the pairs (324,306 pairs) of protein–ligand complexes in the SCOP dataset follow usual assumption that homologous proteins tend to recognize similar ligands with similar binding modes. In this category, 81% of pairs have the identical ligand, and the others are the complexes with analogous ligands. As an example of this category, a superimposed picture of a cytidine deaminase complexed with cytidine and its analogue (PDB IDs: 2FR6 and 1CTT) is shown in Supporting Information Figure S5.

On the other hand, similar binding modes do not always imply similar ligands or similar proteins. Among the 1,940,511 similar binding pairs, 679,040 and 829,303 pairs are with similar ligands and proteins, respectively. The remaining 756,474 pairs have similar binding modes, in spite of the fact that neither the ligands nor the proteins are similar. This is an unprecedented relationship between protein–ligand complexes. Conversely, there are 392,523 pairs with similar proteins and similar ligands, and 68,217 of them have dissimilar binding modes. They are the counter examples of the common sense notion that similar proteins and similar ligands achieve recognition performed with similar binding modes.

The majority of such exceptional relationships are the pairs of frequently appearing ligands, including nucleotides, heme, sugars, cofactors, and their analogues. One of the reasons is the fact that the binding motifs for these well‐known ligands are conserved among a wide range of proteins; e.g., the P‐loop motif recognizing the α‐phosphate of a mononucleotide.^25^ In addition, the PDB is biased toward well‐known ligands: the top 1% high frequency ligands; e.g., heme (PDB three‐letter code: HEM), nicotinamide adenine dinucleotide (NAD), flavin adenine dinucleotide (FAD), and 32 other major ligands, represent 34.6% of the complexes. However, we found some interesting examples that may provide insights into the molecular recognition as discussed below.

### Identity of binding sites in pairs of homologous protein complexes

**Figure 5:**
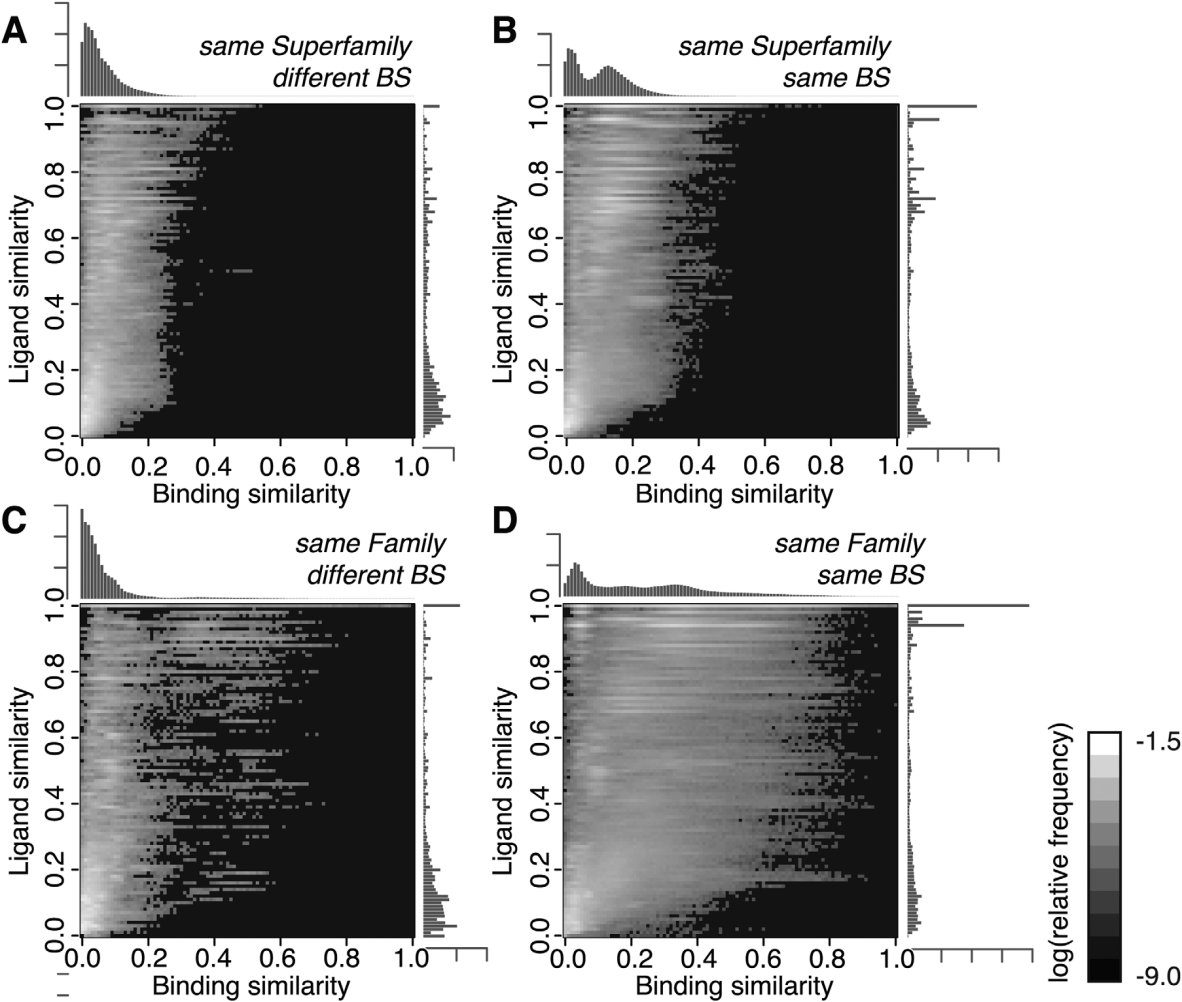
Distributions of ligand and binding similarities in the pairs of same/different binding sites. The histograms of the relative frequencies of pairs over binding and ligand similarities. (A) The pairs of complexes with the same Superfamily and different binding sites. (B) Those with the same Superfamily and the same binding sites. (C) The pairs of complexes with the same Family and different binding sites. (D) Those with the same Family and the same binding sites; see also Figure 2.

In our dataset, 1.9% of pairs (3,673,724 pairs) have similar proteins (pairs are in the same Superfamily/Family). We classified them into the two categories: the pairs with ligands on the same binding site (their ligands overlap in the superimposed picture), and those on the different binding sites. As a result, 81% of them are in the same binding sites. The 2D and 1D histograms of the frequencies of *S*_b_ and *S*_l_ in each combination of the SCOP accordance (Superfamily or Family) and the binding site identity (identical or not) are shown in Figure 5 in the same manner as Figure 2. Their averages and standard deviations are shown in Figure 3 and Supporting Information Table S5.

These figures show clear differences between the pairs with the same binding site and those with the different binding sites. The 1D histograms of *S*_b_ in the pairs with the same binding sites have the second peak at *S*_b_ = 0.13 for the same Superfamily [Fig. 5(B)] and *S*_b_ = 0.33 for the same Family [Fig. 5(D)], in contrast to the fact that the pairs with different binding sites have only one peak at the low *S*_b_ regime. The 2D histograms demonstrate that the distributions widely spread into similar binding modes regime in the pairs with the same binding site. The ligand similarity is also gained in the pairs with the same binding sites compared to those with different binding sites, in particular the pairs of the identical ligand (*S*_l_ = 1.0) are enriched. However, although ligands on the same binding sites of the same Family tends to give high similarity of ligands and binding modes, the correlations between *S*_b_ and *S*_l_ is not so high (*R* ^2^ = 0.509 for the same Superfamily, and *R* ^2^ = 0.415 for the same Family) and there are many pairs with low *S*_b_ and low *S*_l_. This may indicate the presence of low specificity binding sites, which can recognize a variety of ligands in the same binding site.

### Similarity in binding modes between complexes with dissimilar proteins and dissimilar ligands

In the SCOP dataset, 0.38% of the pairs (756,474 pairs) of protein–ligand complexes have similar binding modes with dissimilar proteins and ligands. They include 11,748 complexes consisting of 863 SCOP Families and 2,172 ligands.

**Figure 6:**
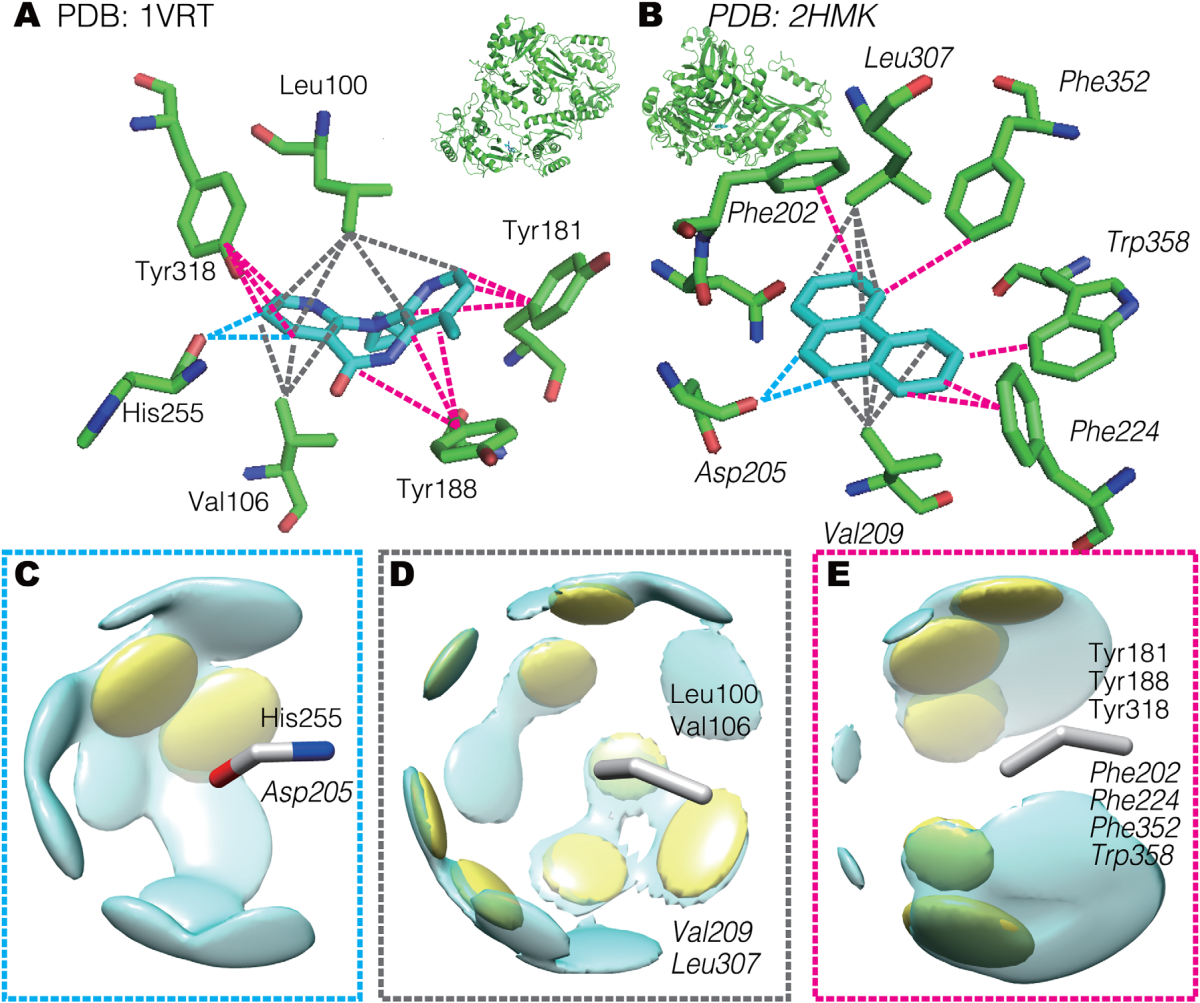
Comparison of binding modes between HIV‐1 reverse transcriptase (PDB ID: 1VRT) and naphthalene 1,2‐dioxygenase (PDB ID: 2HMK). (A,B) Binding structures of 1VRT and 2HMK, respectively. The ligand structure is shown by cyan sticks, and the residues that have common interactions with those in the other complex are shown by green sticks. The ribbons represent the entire structures of the complexes. The dashed lines between a protein atom and a ligand atom indicate common interactions. (C–E) The spatial distributions of the interaction patterns that were commonly applied in these two complexes. The protein fragments; i.e., the successive three atoms in the amino acids, are shown as sticks. The cyan surfaces indicate the overrepresented regions, or interaction patterns, of the locations of ligand atoms interacting with the protein fragment. The yellow surfaces are patterns commonly used in both 1VRT and 2HMK. The interactions shown as cyan, gray, and pink dashed lines in (A) and (B) correspond to those in (C), (D), and (E), respectively. The residue names of 2HMK are labeled as the italic style.

An illustrative example of them is the pair of PDB IDs: 1VRT and 2HMK [Fig. 6(A,B)]. These proteins are in different SCOP Classes; the 1VRT protein is an HIV‐1 reverse transcriptase and its SCOP classification is *e.8.1.2*, which is the reverse transcriptase Family. In contrast, the 2HMK protein is a naphthalene 1,2‐dioxygenase and its SCOP classification is *b.33.1.2*, which is the ring hydroxylating α subunit ISP domain Family. The ligands for 1VRT and 2HMK are nevirapine (PDB 3‐letter code: NVP) and phenanthrene (PEY). Both ligands have a three‐ring scaffold; however, the former has heterocyclic rings with some substituent groups, and its center ring is heptameric. Due to these differences, the fingerprint‐based chemical similarity measure does not detect similarity between them (*S*_l_ = 0.0).

In these binding modes, there are three major types of interactions, which are commonly observed in these two complexes: (i) CH—O weak hydrogen bonds at the backbone of His255 in 1VRT, and at that of Asp205 in 2HMK, (ii) sandwich‐shaped hydrophobic contacts by the two aliphatic residues, namely Leu100 and Val106 in 1VRT, and Leu307 and Val209 in 2HMK, and (iii) several π–π contacts at Tyr181, Tyr188, and Tyr318 in 1VRT, and Phe202, Phe224, Phe352, and Trp358 in 2HMK. They are shown by the (i) cyan, (ii) gray, and (iii) pink lines in Figure 6(A) and (B).

Examples of the interaction patterns shared between the two complexes are shown in Figure 6(C), (D), and (E). For the type (i) interaction, the spatial distribution of the aromatic carbon atoms interacting with a protein fragment consisting of oxygen, carbon, and nitrogen atoms in the amino acid backbone is shown in Figure 6(C). Both His255 in 1VRT and Asp205 in 2HMK interact with two aromatic carbon atoms with the patterns shown by yellow surfaces, and this indicates the presence of bifurcated weak hydrogen bonds between the CH portions and a backbone oxygen atom. For the type (ii) interaction, the distribution of the aromatic carbon atoms of the ligands interacting with the fragment of three sp3 carbon atoms; e.g., the distal side of the side‐chains of valine and leucine, is shown in Figure 6(D). This figure shows that the distribution of the interacting atoms is widely spread in many directions, since this distribution is generated from isotropic, hydrophobic contacts. For the type (iii) interaction, the distribution of the aromatic carbon atoms of the ligands interacting with a fragment composed of three aromatic carbon atoms is shown in Figure 6(E). The interaction patterns are localized over and under the plane of the aromatic fragment, indicating that π–π interactions are favored.

We searched binding sites that are similar to the NVP binding sites in 1VRT, by using two existing binding site similarity search engines, eF‐seek,^26^ GIRAF,^27^ and ProBIS.^28^ However, 2HMK is not detected by these methods (this entry was found in neither top‐100 similar binding sites in eF‐seek and GIRAF, and the Z‐score ≥1.0 hit list in ProBIS). Since the relative positions (both the spatial position and sequence position) of the interacting residues were not similar despite of a high similarity of interactions, these similarity measures could not detect this relationship. Our method can detect such a fuzzy similarity, which does not directly rely on the spatial positions of atoms, due to the graph topology‐based comparison.

### Recognition of similar ligands by similar proteins in different binding modes

We found many examples of cases where similar proteins recognize similar ligands with different binding modes. There are 68,217 pairs (0.04% of the total), including 8,213 complexes, 863 SCOP Families, and 518 ligands. The majority of these cases involve binding with frequently appearing ligands. For example, 37,887 pairs (55.5%) of these cases are the binding sites of heme, and 10,964 pairs (16.1%) are those for NAD. These cases show that two similar ligand molecules can bind to two distinct binding sites on a protein. In some cases, one of the ligands is placed at an interface between symmetric biological units, which may be a result of the crystal packing. For example, PDB ID: 1NKH presents the 3D structure of lactose synthase bound with two uridine 5′‐diphosphate (UDP) molecules for each biological unit (Supporting Information Fig. S6). In comparison with the structure of the same protein in PDB ID: 1PZY with only one UDP molecule, one of the UDP molecules is bound at the identical binding site with a similar binding mode (*S*_b_ = 0.24; *Z*_b_ = 4.6); however, the binding mode of the other UDP, which is at the interface, is very different from them (*S*_b_ = 0.0). The nature of the UDP molecule at the interface between the biological units was not discussed in the original report.^29^

**Figure 7:**
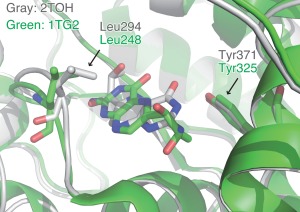
Comparison of the binding modes between tyrosine hydroxylase (PDB ID: 2TOH) and phenylalanine hydroxylase (PDB ID: 1TG2) complexed with 7,8‐dihydrobiopterin. The gray and green molecules indicate 2TOH and 1TG2, respectively. The molecules depicted by sticks at the center of the figure are 7,8‐dihydrobiopterin. The leucine and tyrosine residues, described in the main text, are shown as sticks.

Apart from the frequent ligands, there are 164 pairs with rare ligands, which means only one pair exists among the 68,217 pairs. As an example of them, a tyrosine hydroxylase (PDB ID: 2TOH) and a phenylalanine hydroxylase (PDB ID: 1TG2) complexed with 7,8‐dihydrobiopterin are shown in Figure 7. The resolutions of these X‐ray structures were 2.3Å and 2.2 Å, respectively, and authors mentioned the coordinates of their ligands were readily assigned.^30^, ^31^ The sequence identity of these proteins is 64%, and the SCOP classification is *d.178.1.1*, which is the aromatic amino acid mono‐oxygenases, catalytic, and oligomerization domains Family. Note that although the ligand three‐letter code H2B, which is annotated as the quinonoid 7,8‐tetrahydrobiopterin, is assigned to 1TG2, the authors said that it is 7,8‐dihydrobiopterin in their report.^30^ Interestingly, the orientations of the ligands in the binding sites are opposite between these two complexes. As their aromatic heterocyclic scaffolds are located at almost the same position, their interactions are partially similar (*S*_b_ = 0.102; *Z*_b_ = 1.31). Leu294 in 2TOH and the aligned residue Leu248 in 1TG2 form hydrophobic contacts with the ligand aromatic ring. In contrast, Tyr371 in 2TOH and Tyr325 in 1TG2 recognize their ligands in different ways: Tyr371 in 2TOH hydrogen bonds with the ligand carbonyl oxygen atom by using the distal hydroxyl group of the side‐chain, and Tyr325 in 1TG2 forms hydrophobic contacts with the methyl group of the ligand, by using the aromatic ring of the side‐chain. This example shows that slight changes in homologous proteins can result in drastic alterations of the binding modes.

## Discussion

In this study, we developed a new method for assessing the similarity of binding modes between protein–small ligand complexes. We applied this method for the all‐against‐all comparison of the dataset, and compared the similarity measures for protein structures and those for ligand structures. We then discussed the relationships between the three similarities, the protein similarity *S*_p_, the ligand similarity *S*_l_, and the binding similarity *S*_b_, and provided some examples of interesting pairs of complexes.

Statistically, proteins with the same Fold in the SCOP classification are not highly expected to recognize similar ligands and exhibit similar binding modes even when the ligands are bound into the identical binding site. However, accordance in Superfamily or Family implies similar ligands and binding modes [Fig. 3]. In the same Superfamily, the averages of ligand and binding similarities do not depend on the sequence identity in the range of <90%. Sequence identities ≥90% strongly suggest high ligand and binding similarities (Supporting Information Fig. S4).

We found a certain number of pairs of complexes with interesting properties. There are 756,474 pairs representing cases where dissimilar proteins recognize dissimilar ligands with similar binding modes [Fig. 4]. In addition, 68,217 pairs are cases in which similar proteins recognize similar ligands with distinct binding modes. These two types of disagreements between our binding mode similarity measures and the structure similarity measures illuminate the novel relationships between protein–ligand complexes.

Over the past several decades, the recognition modes of highly frequent ligands, especially nucleotides, have been extensively studied by statistical analyses, and the commonalities of the binding sites among evolutionally distant proteins have been discussed.^5^, ^25^ We focused on the relationships between complexes with uncommon ligands in this study. In the case of the pair of HIV‐1 reverse transcriptase and naphthalene 1,2‐dioxygenase, the clear commonalities in their modes of binding dissimilar ligands are shown in Figure 6; namely, a weak hydrogen bond, hydrophobic contacts by aliphatic side‐chains, and π–π interactions. This example suggests the existence of some universal patterns for specific ligand recognition by proteins, which are sets of interactions commonly observed regardless of protein and ligand structures. In contrast, the other example [Fig. 7] presents the diversity of binding modes of similar proteins and ligands. The binding modes of 7,8‐dihydrobiopterin are in opposite directions between the complexes with the two homologous proteins. This implies that a slight difference in the binding sites can drastically change the binding mode.

Our novel computational technique has revealed these interesting, unprecedented relationships. This is a powerful method to discover knowledge from the rapidly growing structure database, toward understanding the mechanisms of molecular recognition.

## Materials and Methods

### Dataset constructions

We prepared two datasets, the “primary dataset” and the “SCOP dataset”. The former was used to obtain and classify the statistics of atomic interactions (see the next subsection). This dataset was the same as that used in our previous study.^19^ The protein–ligand complexes were collected from the PDB, using the following criteria: (i) the structure was solved by X‐ray diffraction with a resolution ≤2.5 Å, (ii) the protein has ≥30 amino acid residues, (iii) the ligand has ≥6 heavy atoms, and a molecular weight ≥80 and ≤800 Da, (iv) the ligand is not highly exposed to the solvent, which means the relative accessible surface area^32^ of the ligand molecule in the complex form to the isolated form is ≤0.6.

The latter dataset is a subset of the primary dataset. Entries that are not in SCOP ver.1.75,^23^ those complexed with some major nonspecific ligands; e.g., detergents and precipitants (Supporting Information Table S1), and those with a small number of interactions (<10 edges) were excluded. The all‐against‐all comparison was performed with this dataset.

### Classification of atomic interactions

The atomic interactions observed in the primary dataset were classified into patterns, and the interaction patterns were used as labels for the edges of the binding graphs. The classification was performed in the same manner as in our previous study^19^ except for the definition of protein atom types. The atom types were defined by the Tripos force field atom type,^33^ reflecting the topology of each atom, which means that *sp*2, *sp*3, and aromatic carbon atoms are discriminated, for example.

First, the amino acid residues in the dataset were decomposed into fragments, consisting of three covalently‐linked atoms. Second, the contacting pairs of a fragment and a ligand atom were extracted from the complex structures, with a distance threshold of the sum of the van der Waals radii of the atoms and 1 Å as an offset. One contacting pair was described by seven variables: the Tripos force field atom types of four atoms (three protein atoms and one ligand atom), and the 3D Cartesian coordinates of the ligand atom in the local coordinate system, defined on the basis of the protein fragment. This fragment–atom pair is referred to as an “interaction unit” in this report. By gathering the interaction units from all complexes in the primary dataset, the spatial distribution of the ligand atoms around a protein fragment was generated for each type of interaction unit, defined by the combination of the types of the four atoms. For each distribution, the interaction patterns were defined by applying the pattern recognition technique, which infers the parameters of the Gaussian mixture model with the fitness to the given distribution.^34^ The Gaussian mixture model is a linear combination of Gaussian functions,

(2) $$\text{pdf}(x)=\sum_{k=1}^{K}\pi_{k}N\left(x|\mu_{k},\sum_{k}\right),$$

where pdf(**x**) is the probability density function for an observation of a ligand atom at position **x**, which is a local coordinate 3D vector. *π*_k_ denotes a weighting coefficient of the *k*th Gaussian function *N(* ***x*** *|* ***μ***_k_,***Σ***_k_ *)*. ***μ***_k_ and ***Σ***_k_ are the parameters of the *k*th Gaussian function, and are the mean as a 3D vector and the covariance matrix as a positive‐definite, symmetric 3 × 3 matrix, respectively. The maximum number of Gaussian functions in each distribution, *K*, is an adjustable parameter, and we used *K* = 15 to be consistent with our previous study.

When the Maharanobis distance from a data point **x**; i.e., an interaction unit observed in a complex, to an Gaussian function is ≤2.5, this interaction unit is labeled with the ID of the Gaussian function. Only the Gaussian functions assigned with ≥100 data points and with *π*_k_ ≥ 0.1 are regarded as “interaction patterns”.

### Binding graph

The binding graph was generated for each protein–ligand complex in the SCOP dataset, by the following procedure. The 3D structure of a complex was decomposed into a set of interaction units. Amino acid residues (not fragments) and ligand atoms were encoded as nodes, and edges were drawn between pairs with at least one interaction pattern.

The SCOP dataset can be represented as ***G*** = {*g_i_*} (*i* = 1…|***G***|), where *g_i_* denotes the *i*th binding graph, and |***G***| denotes the total number of binding graphs in the dataset ***G***. The graph *g_i_* consists of the set of nodes *V_i_* = {*v_i,m_*} (*m* = 1…*|V_i_|*) and the set of edges *E_i_* = {*e_i,n_*} (*n* = 1…*|E_i_|*). The *m*th node *v_i,m_* has the label *T_i,m_* indicating the type of node, which is one of 20 kinds of regular amino acids or 25 kinds of Tripos force field atom types, for nodes of amino acid residue or ligand atoms, respectively. For the *n*th edge *e_i,n_*, the type of interaction is labeled as a *P*‐dimensional integer vector, ***c***_*i,n*_ = {*c_i,n,p_*} (*p* = 1…*P*), where the number *P* means the total number of interaction patterns defined in the previous subsection. *c_i,n,p_* indicates the number of interactions with the *p*th pattern observed in the edge *e_i,n_*.

As the vector ***c***_*i,n*_ is a raw count of the interaction patterns and it is difficult to directly compare those with different edges, we introduced the normalized interaction vector ***w*** *_i,n_* = {*w_i,n,p_*} (*p* = 1…*P*). The normalization was performed by combining two kinds of measures: (i) the frequency of interaction patterns in each edge and (ii) the frequency of interaction patterns in the dataset. Since the interaction patterns that are ubiquitously observed in a wide range of complexes provide only poor information for characterizing the binding modes, such common interaction patterns were negatively weighted. Each element of the normalized interaction vector *w_i,n,p_* is defined as follows:

(3) $$w_{i,n,p}^{}=\frac{c_{i,n,p}}{\sum_{p^{\prime }}^{P}c_{i,n,p^{\prime }}}\text{log}\left\{\frac{\sum_{i^{\prime }}^{\left|G\right|}|E_{i^{\prime }}|}{\sum_{i^{\prime }}^{\left|G\right|}\sum_{n^{\prime }}^{|E_{i^{\prime }}|}H_{0}\left(c_{i^{\prime },n^{\prime },p}\right)}\right\}$$

where *H*_0_ is the Heaviside step function, which gives 1 only when *c_i,n′,p_* is ≥1 and gives 0 under the other conditions. This is equivalent to the TF–IDF value, which is a well‐known parameter in the field of text‐mining.

### Similarity measure between the binding graphs

The similarity between two binding graphs, *g_i_* and *g_j_*, was calculated in terms of the degree by which the interaction patterns were shared in pairs of aligned edges of the two graphs. The alignment of two graphs was performed, by applying a method for detecting the maximum common edge subgraph (MCES). Detecting the MCES between two graphs is equivalent to detecting the maximum clique of the modular product of the two line graphs.^35^ Since the maximum clique detection is an NP‐hard problem, we applied a heuristics in an iterative manner. This step is illustrated in Supporting Information Figure S1.

To begin with, the similarity between two edges, *S*_e_ *(e_i,n_, e_j,m_)*, was assessed for all possible pairs of *n* and *m*, based on the following definition:

(4) se(ei,n,ej,m)=∑pPmin⁡(wi,n,p,wj,m,p),

Taking the minimum value of the two edges means counting the fractions of the normalized interaction frequency shared by the two edges. Then, the pairs of edges in *g_i_* and *g_j_* were sorted in the descending order of *S*_e_ *(e_i,n_, e_j,m_)*. In the first iteration, only the top *N*_e_ similar edge pairs were picked from the sorted list, and the clique detection algorithm^36^ was applied for the modular product of the two line graphs with the *N*_e_ edge pairs. After that, the next *N*_e_ similar edge pairs were added, and the clique detection was performed again. These processes were iterated until the clique remained unchanged. *N*_e_ is an adjustable parameter, and larger values will provide better results but the computational cost becomes higher. In this study, we used the parameter *N*_e_ = 50, which means that the top 50 most similar pairs of residue‐atom interactions were considered for each iteration step.

After obtaining alignments of the two graphs *g_i_* and *g_j_*, their similarity *S*_b_ *(g_i_, g_j_)* is calculated as follows:

(5) $$S_{b}\left(g_{i},g_{j}\right)=\frac{\sum_{x,x^{\prime }}^{\text{MCES}}s_{e}\left(e_{i,x},e_{j,x^{\prime }}\right)}{\max\left(W_{i,}W_{j}\right)},$$

(6) $$W_{i}=\sum_{n}^{\left|E_{i}\right|}\sum_{p}^{P}w_{i,n,p},$$

where MCES means the set of aligned edges with the running variables *x* and *x′* for the edges of *g_i_* and *g_j_*, respectively. In this definition, *S*_b_ *(g_i_,g_j_)* is within the range of 0 through 1, and *S*_b_ *(g_i_,g_j_)* = 1 means that the binding graphs in *g_i_* and *g_j_* are identical.

In order to evaluate this heuristic approach for the graph alignment, we applied this method to comparing the artificial dataset which is consisting of 160,059 pairs of binding graphs; one of the pair is the binding graph in the SCOP dataset, and the other is a randomly generated subgraph of the former binding graph. As a result, our method correctly aligned all edges for 77.9% pairs, and aligned more than 90% of edges for 80.4% pairs (Supporting Information Fig. S7).

### All‐against‐all comparison of protein–ligand complexes

In the SCOP dataset, an all‐against‐all similarity comparison was performed with the three kinds of similarity measures: the protein similarity *S*_p_, the ligand similarity *S*_l_, and the binding similarity *S*_b_. The protein similarity *S*_p_ was graded in five levels, based on accordance with the SCOP hierarchy; i.e., proteins in the same Family, Superfamily, Fold, Class, and without accordance in any hierarchy. For each pair with the same Superfamily/Family, identity of their binding sites was evaluated. The 3D structures of each pair were superimposed on the basis of their secondary structure elements, by using MICAN.^37^ When the minimum interatomic distance between the ligands is shorter than or equal to 5 Å, this pair is classified as a pair with ligands in the same binding sites.

The ligand similarity *S*_l_ was defined as the Tanimoto coefficient of the chemical structure fingerprints (calculated by the OpenBabel software with the FP2 option^24^). The binding mode similarity was described in the previous subsection.

## Supporting information

Supporting Information
