# Supplementary material for: Landscape of protein–small ligand binding modes
Source: Protein Sci. 2016 Jul 4;25(9):1659–71. doi: 10.1002/pro.2971 (PMC5338237; doi:10.1002/pro.2971)
Supplement: Supplementary file 1 — Supporting Information [file PRO-25-1659-s001.pdf]

Supporting Information for 'Landscape of Protein–Small ligand Binding Modes'

Kota Kasahara[1,*], Kengo Kinoshita[2,3,4]

[1]Collage of Life Sciences, Ritsumeikan University, Kusatsu, Shiga, 545-0021, Japan

[2]Graduate School of Information Sciences, Tohoku University, Sendai, Miyagi 980-8597, Japan

[3]Tohoku Medical Megabank Organization, Tohoku University, Sendai, Miyagi 980-8573, Japan

[4]Institute of Development, Aging and Cancer, Tohoku University, Sendai, Miyagi 980-8575, Japan

[*]To whom correspondence should be addressed

Kota Kasahara

ktkshr@fc.ritsumei.ac.jp

Tel: +81-77-566-1111 Fax: +81-77-561-3729

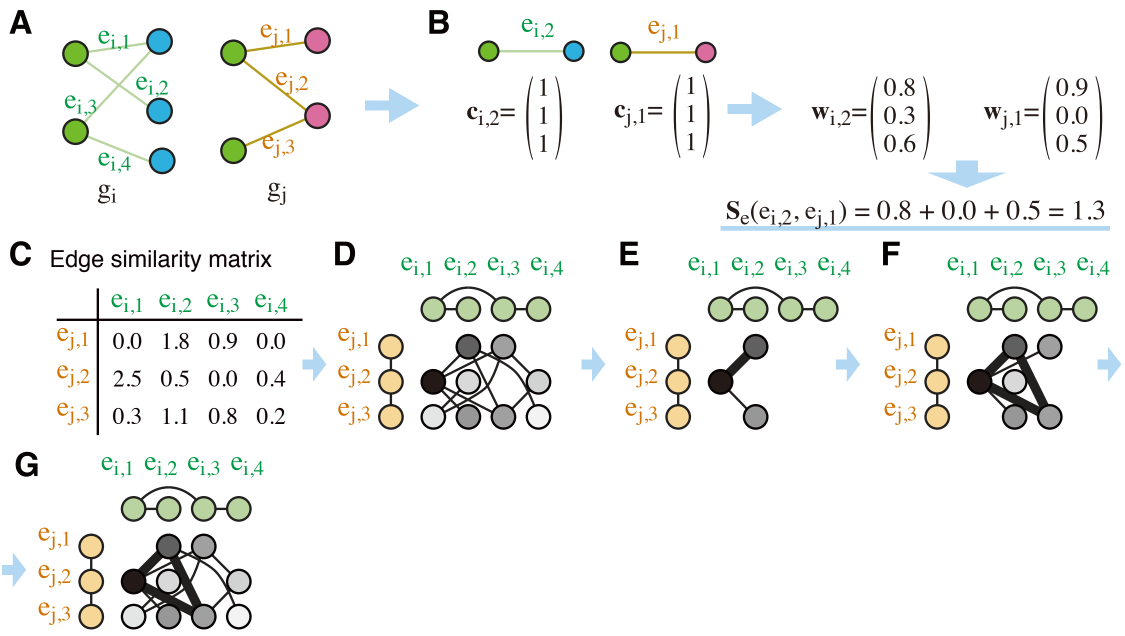

Figure S1 - Details of the similarity assessment between binding graphs

A) Examples of two binding graphs, g_i and g_j. The graph g_i is composed of two amino acid residues (the green circles) and three ligand atoms (the cyan circles) with four interactions (the green lines, labeled $e_{i,1}$, $e_{i,2}$, $e_{i,3}$, and $e_{i,4}$). B) The similarities for all pairs of edges were assessed. As an example, the comparison of the edges $e_{i,2}$ and $e_{j,1}$ is shown. If we assume that there are only three kinds of interaction patterns (P=3), the vectors $\boldsymbol{c}_{i,2}$ and $\boldsymbol{c}_{j,1}$ are 3-dimensional integer vectors with elements that denote the number of each interaction pattern on the edges. These vectors can be transformed into $\boldsymbol{w}_{i,2}$ and $\boldsymbol{w}_{j,1}$ by applying weighting factors. On the basis of these vectors, the similarity between $e_{i,2}$ and $e_{j,1}$ can be calculated as the sum of the smaller values of each dimension in these two vectors. C) The similarities for all pairs of edges can be summarized as a matrix. D) The line graphs of g_i and g_j are constructed, as shown in the top (green) and left (yellow) graphs. The nodes of the line graphs correspond to the edges in the original binding graphs, g_i and g_j. When two edges share a node in a binding graph, an edge is drawn between the corresponding nodes in the line graph. The modular product graph is shown as the grey nodes. A node corresponds to a pair of the line graph nodes, which means a pair of edges in the binding graph. The colors of the nodes in the modular product graph represent the similarities between the corresponding edges, as in the matrix in C). For a pair of nodes in the modular product graph, the edge between them was defined by the corresponding pairs of nodes in the two line graphs. When both the corresponding pairs consistently have (or do not have) their edges, the pair in the modular product graph should have the edge. E) The first iteration in our heuristic alignment method, with N_e=3, is shown. The top three highest

similarity nodes are chosen according to the matrix in C). The maximum clique with the highest similarity was detected (shown as nodes connected with the bold edge). F) In the second iteration, the next top-three nodes are added. The maximum clique was detected again. G) In the third iteration, although the remaining nodes were added, the maximum clique did not change. The alignment of the three pairs of edges in g_i and g_j was obtained as a result of this process.

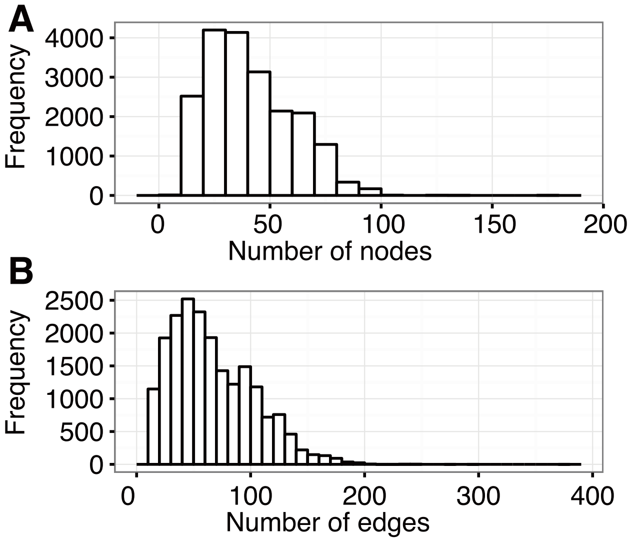

Figure S2 - The distributions of the numbers of nodes and edges in the SCOP dataset

A) The distribution of the number of nodes. B) The distribution of the number of edges.

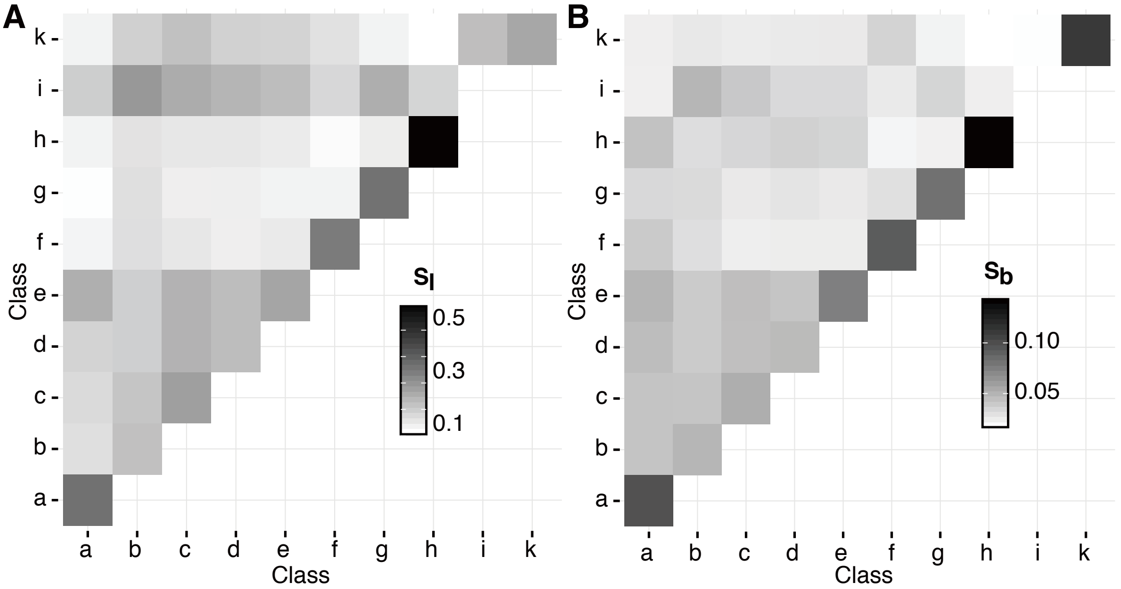

Figure S3 - The average (A) ligand and (B) binding similarities between Classes.

The color of each tile shows the averaged value of the (A) ligand and (B) binding similarities in the pairs of complexes, with the Classes denoted as the horizontal and vertical axes. The numbers of complexes in the Classes are: a=2842, b=3191, c=9346, d=3722, e=560, f=306, g=65, h=3, i=1, and k=4.

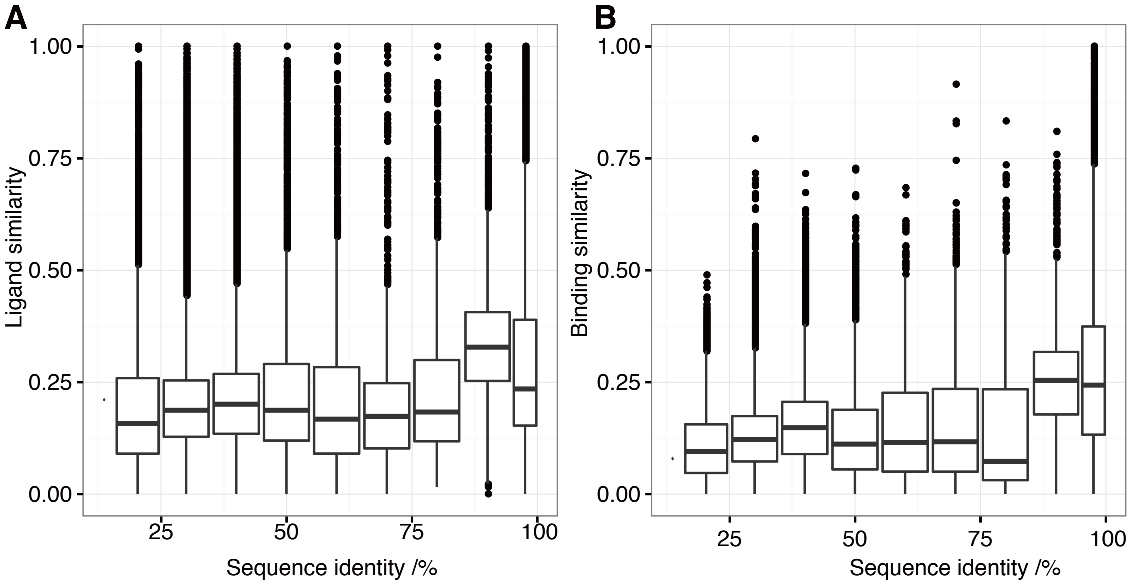

Figure S4 - The distributions of the (A) ligand and (B) binding similarities in each level of sequence identities for the pairs in the same Superfamily.

The horizontal axis is the sequence identity. The vertical axes are the (A) ligand similarity and (B) binding similarity. The center of a box means the median, and the bottom and top of a box mean the first and third quantiles, respectively. The dots are outliers.

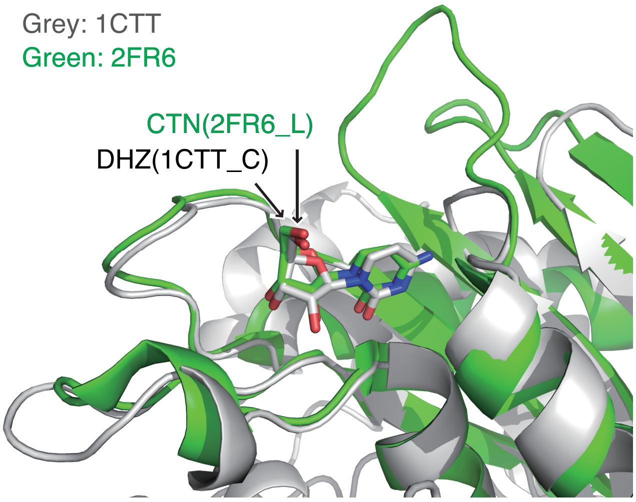

Figure S5 - Cytidine deaminase (PDB IDs: 2FR6 and 1CTT)

The protein structures of cytidine deaminase in 2FR6 and 1CTT are shown as green and grey ribbons, respectively. Their ligands, cytidine (CTN) and its analogue (DHZ) are represented as sticks, and their asym_ids are C and L, respectively.

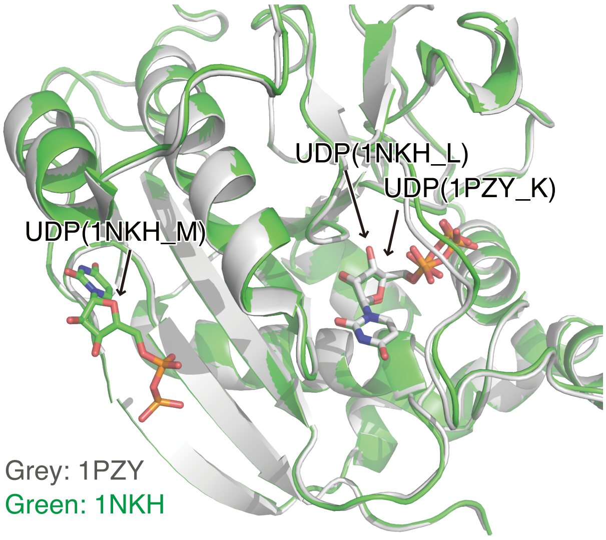

Figure S6 - UDP binding protein (PDB IDs: 1NKH and 1PZY)

The grey and green ribbons represent the protein structures in 1PZY and 1NKH, respectively. The molecule shown as sticks indicates the UDP bound to these proteins. In the structure 1PZY, two UDP molecules are bound (asym_ids, defined in the mmCIF file in the PDB, are M and L). The structure 1PZY has only one UDP molecule (asym id: K). The RMSD value of UDP between 1NKH_L and 1PZY_K is 0.108Å.

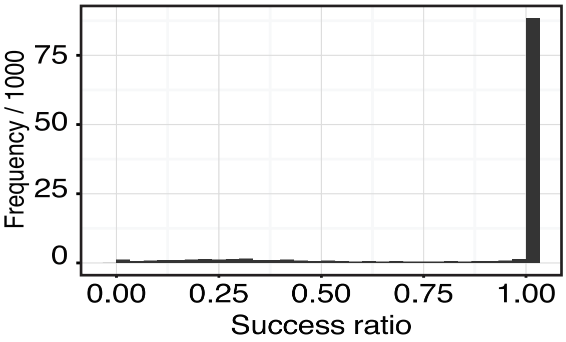

Figure S7 - Test with the artificial dataset

The graph alignment method was tested by using the artificial dataset. An artificial binding graph is generated from a binding graph in the SCOP dataset, by randomly picking a certain amount of connected edges from the original graph. The size of the random subgraph is in the range from 40% to 90% of the original graph. Then, we applied the alignment method to compare between a random subgraph and its original graph. The success rate of an alignment task is measured as ratio of correctly aligned edges.

Table S1 - Statistics of protein fragments

Atom1	Atom2	Atom3	#interactions	#interactions in patterns	Atom1	Atom2	Atom3	#interactions	#interactions in patterns
CAR	CAR	CAR	1,952,083	1,873,686	O3	CAR	CAR	173,014	165,192
C3	C3	C3	1,804,149	1,743,087	NAM	C2	C3	145,526	136,965
O2	C2	C3	814,249	780,971	NAM	C2	O2	145,520	137,443
C3	C3	C2	750,185	710,807	C3	C3	O3	144,512	132,172
C3	C2	O2	730,423	683,943	NAR	CAR	NAR	140,658	130,768
C3	C3	NAM	717,925	680,663	CAR	CAR	O3	122,154	115,140
C3	C2	NAM	624,927	582,840	CAR	C3	C3	116,526	108,925
C2	C3	C3	527,361	493,251	S3	C3	C3	94,871	85,483
O2	C2	NAM	514,783	493,199	CCA	NPL	C3	90,211	80,232
NPL	CCA	NPL	475,198	442,191	N3	C3	C3	84,233	78,415
NAM	C3	C2	428,870	403,566	C3	S3	C3	61,222	55,748
C2	C3	NAM	421,794	392,034	C3	C3	CAR	54,661	48,110
CAR	NAR	CAR	365,529	340,954	NPL	C3	C3	53,144	48,702
C3	NAM	C2	329,149	306,163	C3	NPL	CCA	51,887	45,909
NAM	C3	C3	323,897	303,745	C3	NAM	C3	45,239	40,686
CAR	CAR	C3	318,713	303,996	C3	C3	S3	39,174	33,685
O2	C2	O2	296,403	276,843	C3	C3	NPL	35,164	30,489
O3	C3	C3	252,974	240,844	NAR	CAR	C3	34,026	30,745
NAR	CAR	CAR	240,942	223,763	C3	C3	N3	33,761	29,400
C2	NAM	C3	230,596	214,606	C3	CAR	NAR	26,252	22,549
C3	CAR	CAR	191,858	179,325	S3	S3	C3	5,744	3,517
CAR	CAR	NAR	188,645	170,921	C3	S3	S3	2,505	790

Table S2 - Statistics of ligand atoms

Atom	# interactions	# interactions in pattern
C3	3,818,755	3,653,665
CAR	2,959,426	2,838,083
O3	2,651,525	2,516,623
O2	1,647,263	1,533,116
C2	935,924	886,432
NAR	923,662	872,474
P3	399,999	366,182
NPL	284,008	261,787
S3	174,096	154,651
NAM	142,246	128,271
N3	114,104	100,159
F	41,709	30,892
CL	34,367	25,252
N2	27,920	16,757
S2	18,815	10,287
C1	9,720	3,491
CCA	7,183	2,559
N1	6,646	1,267
N3+	3,522	515

Table S1 - The ligand exclusion list

ALF	CPL	HGX	SF4	EOH	ACA	F23	BOM	EGL	PAM	MA4	TAU	LIO	TAR	SAL
ZPE	MYS	MYR	OHA	PIG	N8E	SPK	PM	IOH	HO2	IOD	SPD	XP4	URE	MRY
XPM	CYN	O2U	XPE	GPX	MRD	1WV	PG5	BNG	6JZ	IPA	9HO	PL3	LAK	IPH
SYL	DTT	DR6	13S	CXE	G2A	LAT	PYR	IHD	CMO	DMF	STE	1EX	GBL	CRY
ICI	LMU	MES	OPC	DMS	DMU	7PE	DMX	78M	FLC	BEQ	PCF	NO3	EPE	LDA
SCN	ELD	6OB	13R	LSM	YT3	8PE	M2M	B8M	P15	GOG	DDQ	ERU	BU1	PEG
GCD	PX4	OLA	BU2	BU3	MN3	B3P	PGE	MHA	4R8	DLP	CE1	PC1	TRS	7E8
TRT	F15	HTG	MPG	MPD	PIN	ODD	HTO	MPO	TBU	1QW	CBM	MOH	BTC	BTB
CBX	BE7	BCN	UMQ	DXG	GLO	P4G	PEU	IDT	SUL	P4C	ACN	DOX	14V	ETF
ACT	14U	ACY	2OS	NER	7E9	OLB	OLC	OP2	CU1	16D	FCY	ASY	ODT	MLI
T24	243	LNL	FE2	16Y	16P	TEP	P33	PCW	FCL	1BO	CLO	UNX	ELA	HEZ
PDO	TLA	PC9	JEF	1PE	UNL	AE4	HXA	CO3	GP7	PE8	SBT	PE4	PE7	LMT
EDO	L9R	BRO	OXY	B9M	P3G	PSC	PLM	AZI	DIO	DPR	12P	VCA	DIA	PC7
RCL	EEE	EIC	RCO	C8E	P6G	CIT	CAC	1PS	C10	MC3	78N	C15	TOE	NHE
MG8	1EY	PGO	GYM	TMA	POP	POL	SOR	PGR	GAI	PGQ	B7M	R16	FMT	GOL
144	6OC	1PG	PO4	DHD	HGP	3NI	PG6	NH4	PG4	BMA	SO4	MTL	B7G	CM5
ICT	ETE	15P	TRE	SDS	MBE	4HD	OTE	1AG	MVC	NCO	IDO	TFP	4E6	

Table S4 - Summary of datasets

Dataset	Primary dataset	SCOP dataset
PDB entries	23,040	9,224
Binding sites	66,654	20,040
Ligand variations (three-letter ID)	6,747	3,507
Interacting protein fragment types	80	63
Ligand atom types	25	22
Elemental interaction types	955	879
Interacting amino acid residues	832,981	325,551
Ligand atoms	1,332,555	538,557
Interacting ligand atoms	1,179,357	487,132
Elemental interactions	14,201,211	9,843,664
Interaction patterns	5,497	5,497
Protein fragment types in patterns	44	44
Ligand atom types in patterns	19	18
Elemental interaction types in patterns	539	494
Interacting residues in patterns	829,312	324,097
Interacting ligand atoms in patterns	1,175,987	486,733
Elemental interactions in patterns	13,402,463	5,608,656

Table S5 - Averages and Standard Deviations of Binding and Ligand Similarities

SCOP accordance	$\mu(S_b)$	$\sigma(S_b)$	μ (S_l)	σ (S_l)	R^2	# pairs
None	0.0424	0.0315	0.166	0.156	0.237	127,416,592
Class	0.0495	0.0371	0.219	0.205	0.356	68,058,614
Fold	0.0440	0.0354	0.160	0.133	0.204	1,641,850
Superfamily	0.0981	0.0762	0.478	0.349	0.504	1,947,199
Family	0.217	0.191	0.547	0.383	0.443	1,726,525
Superfamily with different BSs	0. 0637	0.0548	0.350	0.293	0.362	453,099
Superfamily with the same BS	0.108	0.0786	0.516	0.356	0.509	1,494,100
Family with different BSs	0.0709	0.0991	0.297	0.306	0.367	231,422
Family with the same BS	0.239	0.1917	0.585	0.379	0.415	1,495,103
All pairs	0.0469	0.0420	0.190	0.187	0.307	200,790,780

μ and σ indicate the average and standard deviation of the similarity values. BS stands for "binding site".
